# 

**Published:** 1895-08-24

**Authors:** 


**Notices of Births, Deaths, and Marriages** are inserted in this column at a charge of 2s. 6d. for an announcement not exceeding four lines.

**Notice to Advertisers and Subscribers.**

All Advertisements, Orders for Copies of The Hospital and Business Communications should be addressed to **The Manager, NOT TO THE EDITOR,** The Hospital, 428, Strand, London, W.C.

The Cost of Subscription, post free, is as follows:—Per week, 2½d.; per quarter, 2s. 8d.; per half-year, 5s. 3d.; per annum, 10s. 6d. Foreign:—Per Annum, 15s. 2d.; payable, in all cases, in advance.

### NOTICE TO CORRESPONDENTS.

All MS., Letters, Books for Review, and other matters intended for the Editor should be addressed The Editor, The Lodge, Porchester Square, London, W.

The Editor cannot undertake to return rejected MS., even when accompanied by stamped directed envelope.

**"Burdett's Hospital Annual," 1895.**

Sixth year of publication. 950 pp. Scarlet cloth, gilt lettered. Price 5s. A most complete guide to British, Colonial, Indian, and American Hospitals, Dispensaries, Nursing and Convalescent Institutions, and Asylums. A few copies of the "Annual" for 1894 may still be obtained on application to the Publishers, The Scientific Press (Limited), 428, Strand, London, W.C.

**NOTICE.—Vol. XVII. of "The Hospital" (October, 1894, to April, 1895), in handsome cloth binding, is on sale at the Office of this Journal. Price 6s. Cases for binding the Numbers contained in this Volume, 1s. 6d. Index to Vol. XVII., 2½d., post free.**

**The Monthly Part for August is now ready, price 6d.; by post, 8d.**

### SCRAPS AND GLEANINGS.

Mr. Hughes has been appointed Inspector of Nuisances by the District Council at Bilston.

The City ball held at Liverpool in honour of the convalescent institution, brought in £113 to its funds.

The site for the new infectious diseases hospital for the burgh of Fife is near to Smeaton, on the Dunnikier Estate.

A superannuation allowance of £30 per annum has been granted to Mr. T. H. Bishop, late vaccination officer of the Dorchester Union.

At Glasgow the proceeds of the sale of some particularly pretty election badges were given to the funds of the Hospital for Sick Children in that town.

The Kettering Rural District Council have made an application for sanction to borrow £2,500 for the erection of an isolation hospital at Kettering for the district.

As compared with the first half of 1894, the present year of the Norfolk and Norwich Hospital shows in donations a decrease of £220, and in subscriptions of £122.

The hospital ship at Hull stands in mid-stream, and is in charge of a doctor and matron. It is used exclusively for the isolation of cases of infectious diseases occurring on vessels.

Sanitary authorities continue to urge that the Salvation Army shelters should be placed by Government under the Common Lodging House Act, many cases of small-pox having developed in these places.

Photographs are said to form valuable evidence in court of conditions in existence when medical officers of health visit places where such conditions, although removable, are recurrent.

A successful issue attended the Hospital Sunday demonstration in the Branksome district, held under the auspices of the Parkstone, Heatherlands, Newtown, and Bourne Valley United Hospital Committee.

The habitual casual is said, by those who know him, to be remarkably clean, owing to the number of baths made compulsory for him, although his clothes, face, and hands may give a contrary impression to the ordinary observer.

The great increase of measles in the East-end of London has given rise to a suggestion that the disease should be made notifiable as an infectious disease. This step has been taken by other sanitary authorities with good results.

The annual demonstration organised by the various friendly and trade societies of Oxford, on behalf of the Radcliffe Infirmary, has just taken place. A collection in aid of the local charities was made in the cathedral, after a special service.

At a church parade of the Bolton Volunteers held on the 11th inst. Mr. James Briscoe, secretary to the infirmary and dispensary, was the recipient of the Volunteer Long Service Medal. He wore his Crimean medals on the occasion.

The late Mr. Joseph Huntley has left about £10,000 for distribution between the Peace Society, the Friends' Foreign Missionary Society, the Royal Berkshire Hospital, the Reading Dispensary, the Temperance Society, the British Schools, &c.

Hospital Saturday at Swansea shows in financial results a short decrease this year, as compared with last year's receipts. One feature of the year's record of the Swansea General Hospital is the fact that the workmen's subscriptions have increased from £689 to £1,028.

The fifth annual international congress combating the abuse of alcoholic liquors was arranged in Bale for the last week in August. Amongst the papers contributed on the occasion nine, devoted to the physiological aspects of alcoholism, were by celebrated physicians and professors.

Dr. Legrain, physician of the Paris Lunatic Asylums, has recently founded the first French Temperance Society, of which the pledge stands "I promise firstly to abstain, except on medical advice, from brandy and all liqueurs; secondly to make use only in moderation of wine, beer, or cider."

The 157th annual report of the Royal Hants County Hospital has just been presented. The number of patients under treatment during the past year has been 1,154. The committee, unfortunately, have cause to regret that their financial statement compares unfavourably with those of recent years.

The question of the new epidemic hospital at Arbroath being managed under the direction of the existing infirmary does not seem to be settling itself. "This infirmary," a local paper remarks, "would, we believe, still further popularise itself as an important healing institution if it were to undertake the management of the epidemic hospital."

Cookery, domestic economy, needlework, and dressmaking are attracting increased attention under the London Technical Education Scheme. From the comments of the examiners in these subjects, reported in the *London Technical Education Gazette*, better instruction in all these subjects appears most desirable for the girls.

The Children's Convalescent Home at Croydon is maintaining its standard of useful work. No less than 138 children have been admitted during the past twelve months. The total receipts of the year have been brought up to £386, but this sum is insufficient to cover the working expenses for the year, and an adverse balance, though a very small one, is showing signs of increase.

Mr. William Berry has bequeathed £10,000 to the Manchester Infirmary, £5,000 to the Manchester City Mission, £3,000 to the Boys' Refuge, £2,000 to the National Lifeboat Institution, £2,000 to the Southport Convalescent Hospital, £1,000 to Dr. Barnardo's Homes, £1,000 to the Royal Eye Hospital, £1,000 to the Old Trafford Blind Asylum, and £1,000 to the Prevention of Cruelty to Children Society.

Miss Donald McFee, of Montreal, has taken the degree of PhD. at the University of Zurich. She is a B.A., graduate in honours of McGill University, Montreal, and took a post graduate course of philosophy at Cornell University, U.S., afterwards studying for two years at Leipzic under Dr. Wundt, and finally taking the full doctoral course required at Zurich for the degree which she has recently distinguished herself by taking.

### THE STUDENT OF MEDICINE.

### APPOINTMENTS.

A. E. J. Barcroft, L.R.C.S.I., L.K.Q.C.P.I., appointed Medical Officer for the Myddle and Baschurch District of the Ellesmere Union. Dr. Beaumont, appointed Medical Officer for the Camberwell Union Infirmary. N. T. Bond, M.B., C.M.Edin., appointed Medical Officer for the No. 4 District of the Liskeard Union. J. N. Bredin, L.R.C.S.I., L.K.Q.C.P.I., L.S.A., appointed Medical Officer for the Potton District of the Biggleswade Union. W. Carey, L.R.C.S.I., L.M., appointed Medical Officer for the Workhouse and the Holbrook District of the Samford Union. R. Coates, L.R.C.P., M.R.C.S., appointed Resident Medical Officer to the Newport and Monmouthshire Infirmary. C. P. Crouch, M.B.Lond., F.R.C.S.Eng., appointed Honorary Surgeon to the Weston-super-Mare Hospital. Dr. J. C. Fletcher, appointed Medical Officer and Vaccinator for the Winster District of the Bakewell Union. Mr. E. F. Green, appointed Medical Officer for the No. 2 District of the Steyning Union. A. Gregor, M.B., appointed Medical Officer for the Sutton-on-Trent District of the Southwell Union. J. L. Johnstone, L.R.C.P.Lond., M.R.C.S.Eng., appointed Medical Officer for the Upholland District of the Wigan Union. Mr. D. W. Jones, appointed Medical Officer for the Inkberrow District of the Alcester Union. Dr. R. D. Jones, appointed Medical Officer for the Grangetown District of the Cardiff Union. W. R. Kingdon, M.B.Durh., appointed Junior Resident Medical Officer to the Stoke Newington Dispensary, London, N. G. G. Parsons, M.B., appointed Medical Officer for the Fourth District of the Westbury and Whorwellsdown Union. Dr. J. J. Powell, appointed Medical Officer for the First District of the Highworth and Swindon Union. Mr. F. W. Reilly, appointed Medical Officer for the Bellinge District of the Wigan Union. T. Rhind, M.R.C.S.Eng., L.R.C.P.Lond., appointed Medical Officer for the No. 1 Western District of the Billesdon Union. A. H. Roberts, L.R.C.P.Lond., M.R.C.S.Eng., appointed Medical Officer of Health to the Malling Rural District Council. W. A. S. Royds, L.R.C.P.Lond., M.R.C.S.Eng., appointed Medical Officer for the St. Mary Bowine District of the Whitchurch (Hants) Union. G. C. Sandford, M.B., appointed Resident Obstetric Surgeon to the Dispensary, York. E. C. Stubb, F.R.C.S., appointed Assistant in the Throat Department at St. Thomas's Hospital. Dr. Thompson, appointed Medical Officer for the Twycross District of the Market Bosworth Union. Mr. C. E. Weeks, appointed Medical Officer for the Pinchbeck District of the Spalding Union. E. M. Wyche, M.R.C.S., L.R.C.P., appointed Assistant Medical Officer to the Infirmary of the City of London, Bow Road, E.

### VACANCIES.

The following vacancies are announced this week, the amount of salary in each case being given after the name of the institution:—

#### Resident Medical Officers.

Cumberland Infirmary, Carlisle.—House Surgeon. Salary £70 per annum, with board, lodging, and washing. Appointment for one year. Applications to the Secretary by August 24th.

General Hospital for Sick Children, Pendlebury, Manchester.—Junior Resident Medical Officer, doubly qualified; must devote his whole time. Salary £80 per annum, with board and lodging. Appointment for one year. Applications to the Chairman of the Medical Board by August 27th.

General Hospital, Nottingham.—House Physician. Appointment for two years, but eligible for re-election. Salary £100 per annum, rising £10 a year to £120. Assistant House Surgeon. Appointment for six months. Board, lodging, and washing in hospital; no salary. Applications to the Secretary for the former post by September 11th, and for the latter by September 7th.

Jaffray Suburban Branch of the General Hospital, Gravelly Hill, Birmingham.—Resident Medical and Surgical Officer, doubly qualified. Salary £150 per annum, with board, residence, and washing. Applications to the House Governor, General Hospital, Birmingham, by August 24th.

Norfolk and Norwich Hospital.—Assistant House Surgeon, doubly qualified. Appointment for six months. Board, lodging, and washing provided. Applications to the House Surgeon by September 2nd.

Parish of Lambeth.—Assistant Medical Officer and Dispenser for the Infirmary in Brook Street, Kennington; doubly qualified. Must devote his whole time to the duties of his office. Salary £125 per annum, with board, furnished apartments, and washing. Applications, on forms provided, to W. B. Wilmot, Clerk, Guardians' Board-room and Offices, Brook Street, Kennington Road, S.E., by August 24th.

Royal Southern Hospital, Liverpool.—Third House Surgeon. Salary £65 per annum, with board, lodging, and laundry. Applications to the Chairman of the Medical Board by August 26th.

#### Non-Resident Medical Officer.

School Board for London.—Medical Officer for the Board's Training Ship *Shaftesbury*, lying off Grays, Essex; must reside within two miles of the ship. Commencing salary £100 a year, which may be increased by annual increments of £10 to £150 a year. Applications to A. E. Garland, Clerk to the Managers, School Board Offices, Victoria Embankment, W.C., by August 31st.

### The Victoria University.

Pass Lists: Faculty of Medicine—Final Examination.

Part I.—G. Ashton, Owens College; W. S. Badger, University College; F. J. Batten, Owens College; R. Bleasdale, Owens College; H. M. Brown, Owens College; A. H. Burgess, Owens College; J. B. Clarke, Owens College; H. Coates, Owens College; C. Coventry, Owens College; H. M. Crake, University College; P. H. Fearnsides, Yorkshire College; H. Hamer, Owens College; E. Hutton, Owens College; W. F. Jackson, Owens College; K. H. Jones, Owens College; A. T. Lakin, Owens College; J. G. Martin, University College; E. Monks, Owens College; N. Neild, Owens College; W. H. S. Nickerson, Owens College; A. E. Normington, Owens College; J. S. Ross, Owens College; S. T. Rowling, Yorkshire College; E. A. Smith, University College; L. Tong, Owens College; A. A. Wood, University College; J. Wood, Owens College; H. B. Woodcock, Owens College; H. C. Woodhouse, Owens College.

Part II.—A. V. Davies, Owens College; U. L. Desai, Owens College; A. Greenwood, Owens College; R. S. Hardman, Owens College; F. B. G. Holmes, Owens College; C. E. Ligertwood, Yorkshire College; J. P. Lowe, Owens College; A. McDougall, Owens College; J. T. Marsden, Owens College; T. H. Miller, Owens College; F. C. Moore, Owens College; J. H. Renshaw, Owens College; J. V. Shaw, Yorkshire College; R. H. Trotter, Yorkshire College; B. V. Watkins, Owens College.

The following have been awarded honours:

Class I.—C. E. Ligertwood, Yorkshire College; F. C. Moore, Owens College.

Class II.—R. S. Hardman, Owens College; F. B. G. Holmes, Owens College; T. H. Miller, Owens College; J. H. Renshaw, Owens College; J. V. Shaw, Yorkshire College; R. H. Trotter, Yorkshire College; B. V. Watkins, Owens College.

Diploma in Sanitary Science.—M. Parkinson, F. W. Sydenham.

